# A PILOT ASSESSMENT OF A TABLET-BASED INTERVENTION FOR HOMEBOUND OR SOCIALLY ISOLATED OLDER ADULTS WITH DEPRESSION

**DOI:** 10.1093/geroni/igac059.3135

**Published:** 2022-12-20

**Authors:** Caroline Galo, Natalie Benda, Isabel Olivia Rollandi, Sara Czaja, Marco Ceruso, Jo Anne Sirey

**Affiliations:** Weill Cornell Medicine, New York, New York, United States; Weill Cornell Medicine, New York, New York, United States; Weill Cornell Medicine, White Plains, New York, United States; Weill Cornell Medicine, Ithaca, New York, United States; Weill Cornell Medicine, New York, New York, United States; Weill Cornell Medicine, White Plains, New York, United States

## Abstract

Older adults who are homebound or socially isolated have high rates of loneliness and depression with fewer opportunities for treatment. Our team extended an existing psychotherapy intervention (Engage & Connect) to improve access to mental health care for older adults who are homebound. We iteratively created a tablet-based application (Engage PRISM) leveraging a user-centered design approach to provide older adults, particularly those with limited technology experience, an easy-to-use application to support social reward. Engage PRISM connects clients with the psychotherapy intervention and additional features to increase social reward exposure virtually. All eligible participants received a K92 ZTE tablet, equipped with the Engage PRISM application, internet service, Zoom, and access to Selfhelp’s Virtual Senior Center. Participants were then enrolled in the 9-week Engage & Connect intervention delivered by a licensed mental health counselor via Zoom on the tablet each week. We evaluated feasibility of the intervention and preliminary effect on depressive symptoms through a weekly PHQ-9. Feasibility was assessed through participants’ ability to use the tablet to access mental health treatment. We provided tablets to eight participants ages 67 to 84; participants demonstrated 100% feasibility of use of the tablet intervention. We dropped two participants from the study due to a greater level of care needed. All participants were provided with referrals prior to ending the study. Preliminary evidence indicates that four of the six remaining participants had experienced a reduction in depressive symptoms (i.e., had lower PHQ-9 scores) three weeks into the study reporting over a 30% reduction on average.

